# Long‐term dynamics of a cladoceran community from an early stage of lake formation in Lake Fukami‐ike, Japan

**DOI:** 10.1002/ece3.7112

**Published:** 2020-12-24

**Authors:** Yurie Otake, Hajime Ohtsuki, Jotaro Urabe, Shigeko Kimura, Kazuyoshi Yamada, Takehito Yoshida

**Affiliations:** ^1^ Department of General Systems Studies University of Tokyo Meguro Japan; ^2^ Ecology and Evolutionary Biology Graduate School of Life Sciences Tohoku University Sendai Japan; ^3^ School of Environmental Science The University of Shiga Prefecture Hikone Japan; ^4^ Museum of Natural and Environmental History Shizuoka Japan; ^5^ Faculty of Human Sciences Waseda University Tokorozawa Japan; ^6^ Research Institute for Humanity and Nature Kyoto Japan

**Keywords:** cladoceran, eutrophication, long‐term change, paleolimnology, varved sediment

## Abstract

An increase in nutrient levels due to eutrophication has considerable effects on lake ecosystems. Cladocerans are intermediate consumers in lake ecosystems; thus, they are influenced by both the bottom‐up and top‐down effects that occur as eutrophication progresses. The long‐term community succession of cladocerans and the effects cladocerans experience through the various eutrophication stages have rarely been investigated from the perspective of the early‐stage cladoceran community assemblage during lake formation. In our research, long‐term cladoceran community succession was examined via paleolimnological analysis in the currently eutrophic Lake Fukami‐ike, Japan. We measured the concentration of total phosphorus and phytoplankton pigments and counted cladoceran and other invertebrate subfossils in all layers of collected sediment cores, and then assessed changes in the factors controlling the cladoceran community over a 354‐year period from lake formation to the present. The cladoceran community consisted only of benthic taxa at the time of lake formation. When rapid eutrophication occurred and phytoplankton increased, the benthic community was replaced by a pelagic community. After further eutrophication, large *Daphnia* and high‐order consumers became established. The statistical analysis suggested that bottom‐up effects mainly controlled the cladoceran community in the lake's early stages, and the importance of top‐down effects increased after eutrophication occurred. Total phosphorus and phytoplankton pigments had positive effects on pelagic *Bosmina,* leading to the replacement of the benthic cladoceran community by the pelagic one. In contrast, the taxa established posteutrophication were affected more by predators than by nutrient levels. A decrease in planktivorous fish possibly allowed large *Daphnia* to establish, and the subsequent increase in planktivorous fish reduced the body size of the cladoceran community.

## Graphical abstract

In this research, we examined the long‐term changes of a cladoceran community from the early stage of lake formation by using paleolimnological methods. Our results suggested that the cladoceran community firstly consisted only of benthic taxa, and subsequent rapid eutrophication caused the replacement of benthic community species to planktonic pelagic ones. In addition, artificial introduction of fish possibly caused the change of predator community, leading to the increase in top‐down effect on the cladoceran community.

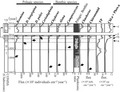

## 1 INTRODUCTION

Community assembly and long‐term dynamics are central topics in ecology. Most lakes are formed under oligotrophic conditions, and eutrophication gradually occurs even without human activity (Sakamoto, 1973; USEPA, 2009). Increasing nutrient levels have important effects on the succession of lakes and their ecosystems. In a lake ecosystem, long‐term succession with increasing nutrient levels has been researched through long‐term observations comparing multiple lakes (Vadeboncoeur et al., 2001, 2003) and paleolimnological analysis that reconstruct long‐term changes using lake sediments and subfossils (Moss, 1979; reviewed in Davidson & Jeppesen, 2013). These previous studies mainly focused on the changes in lake ecosystems from recent industrial eutrophication, which has been rapidly occurred on a global scale from the 1950s to 1960s (Schindler, 2006) and has become a major environmental problem affecting water systems (Smith & Schindler, 2009; USEPA, 2009). Eutrophication can cause the dominant primary producers to change from submerged plants to pelagic algae, leading to shifts in the principal location of primary production from the benthic to the pelagic zone (Moss, 1979; Sayer et al., 2010; Vadeboncoeur et al., 2001, 2003). In addition, excess nutrients cause changes in fish communities, and an increase in total phosphorus (TP) leads to an increased relative abundance of planktivorous fish to piscivorous fish, resulting in the intensification of predation risk to zooplankton (Jeppesen et al., 1997).

Since cladocerans are intermediate consumers in lake food webs, they constitute an important group that links primary producers with high‐order consumers, and the cladoceran community is affected by both bottom‐up and top‐down effects (Carpenter & Kitchell, 1993). Some parts of the cladoceran body (e.g., postabdominal claw, carapace, and head shield) are composed of chitin, and they are well preserved over the long term from one to several thousand years (Korhola & Rautio, 2001; Szeroczyńska, 1991). Thus, cladoceran community dynamics affected by eutrophication were analyzed by the paleolimnological method comparing the layers of lake sediment before and after eutrophication had occurred (e.g., Bennion et al., 2015; Ohtsuki et al., 2015; Taylor et al., 2006 and more). This paleolimnological analysis and other long‐term observations revealed the following changes in the cladoceran community caused by eutrophication. First, the cladoceran community changed from a benthic to a pelagic community by sensitively responding to the benthic to pelagic shift in primary production (Bennion et al., 2015; Davidson et al., 2011; Jeppesen et al., 2011; Taylor et al., 2006). Second, the relative importance of top‐down effects increased compared with bottom‐up effects on the cladoceran community (McQueen et al., 1986) through predation risk from planktivorous fish, which increased with eutrophication (Davidson & Jeppesen, 2013; Jeppesen et al., 1997). This resulted in the decrease in body size of cladocerans (Jeppesen et al., 2001).

Based on these previous studies, we proposed a hypothesis about sequence cladoceran community dynamics from the early stage of lake formation. In the early stage of lake formation, when the nutrient condition is oligotrophic, benthic cladocerans are introduced and form a community. As eutrophication proceeds, this benthic community is replaced by a pelagic community. Further increases in nutrient levels release the cladoceran community from bottom‐up control and allow high‐order consumers to become established. As a result, the importance of top‐down effects on the cladoceran community increases significantly.

However, continuous community succession and changes in the relative effect of bottom‐up and top‐down effects from an early stage of cladoceran community assembly during a lake formation period have rarely been studied; therefore, few previous studies have tested this hypothesis. Furthermore, only a few previous studies have observed the early stage of cladoceran community assembly in newly formed lakes (e.g., Allen et al., 2012). This scarcity is due to difficulties in long‐term observations of lake systems. Paleolimnology has solved such difficulties by allowing the reconstruction of past change (Douglas, 2013; Smol, 2010). Prior paleolimnological studies that analyzed long‐term cladoceran community change and related factors can broadly be divided into two types based on their observation periods. One major type of study compared cladoceran communities between before and after a marked environmental change (e.g., eutrophication: Davidson et al., 2011; acidification: Nevalainen, Sarmaja‐Korjonen, et al., 2011; industrial development: Nevalainen, Luoto, et al., 2011; artificial fish introduction: Strock et al., 2013; reduction of Ca and introduction of copper sulfate: Korosi & Smol, 2012c). Most of these studies observed changes over less than a 100‐year period. Second, some studies have examined changes over thousands of years. For example, a study examined the response of a cladoceran community to climate change in the Holocene until 4,500 cal BP (Nevalainen et al., 2015); another study examined the effects of human activity on a cladoceran community over more than 10,000 years, dating back to the prehistoric period (Szeroczyńska, 1991). However, lakes from which sediment core samples including the lake formation period can be taken are very rare, and most lakes that preserve high‐quality sediment cores are ancient. Thus, even those studies investigating changes over 10,000 years have not analyzed sediments from the lake formation period.

Previous studies analyzed cladoceran community formation by observing the cladoceran community in a water column for 3 years (Louette et al., 2008) and by examining sediment core samples from a new artificial lake, which formed only decades ago (e.g., Allen, VanDyke, & Cáceres, 2011). However, these studies could not investigate the long‐term changes in the controlling factors of environmental effects due to the short research periods. On the other hand, other studies have compared cladoceran communities at several time points over a long‐term eutrophication process (e.g., Straile & Geller, 1998). Such studies can evaluate the cladoceran community response to eutrophication but cannot assess changes that occurred in the early stage of community formation. In addition, although some previous studies have examined the temporal transition from bottom‐up to top‐down effects on cladoceran communities using a paleolimnological method, these studies did not examine effects on entire cladoceran communities. For example, Perga et al. (2010) examined bottom‐up and top‐down effects on only *Daphnia* and *Bosmina*, and Nevalainen et al. (2017) assessed the effects on only one functional group of cladoceran.

In this study, we successfully collected lake sediment cores that included the lake formation period and analyzed changes within a cladoceran community from the early stage of lake formation via paleolimnological analysis. Our study site was Lake Fukami‐ike in Nagano Prefecture, Japan. Lake Fukami‐ike is shallow and surrounded by mountains (maximum depth: 7.8 m; Yagi et al., 2009) with anoxic hypolimnion from April to November (Yagi et al., 1983); thus, there is little disturbance from winds and benthos. Varved sediment core samples had been collected in prior studies on this lake (Kawakami et al., 2004). Varved sediments comprise annual coupled layers forming annual lamina, and allowed us to observe high‐resolution temporal changes (Lamoureux, 2001). Therefore, sediments from Lake Fukami‐ike allowed us to analyze the high‐resolution temporal changes from the lake formation period. Thus, in the present study, we attempted to reconstruct the continuous succession process of a cladoceran community from lake formation to test whether the assumed change in bottom‐up and top‐down effects had occurred.

## 2 MATERIALS AND METHODS

### 2.1 Sediment core sampling

**Figure 1:**
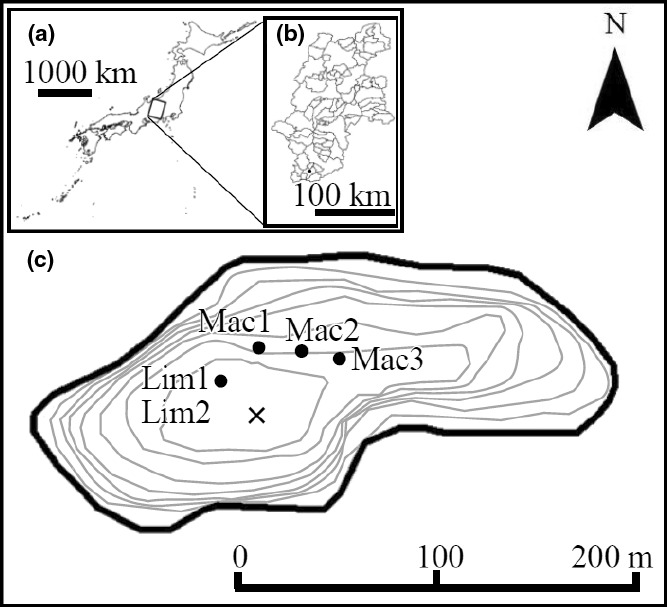
Map of Lake Fukami‐ike (C, 35°19′N, 137°49′) showing the sites from which sediment core samples were taken. The lake is located in Nagano Prefecture (b) in central Japan (a). Lim1 and Lim2 indicate sites where the sediment core samples were taken by a Limnos corer, and Mac1 to Mac3 indicate those taken by a Mackereth corer

**Figure 2:**
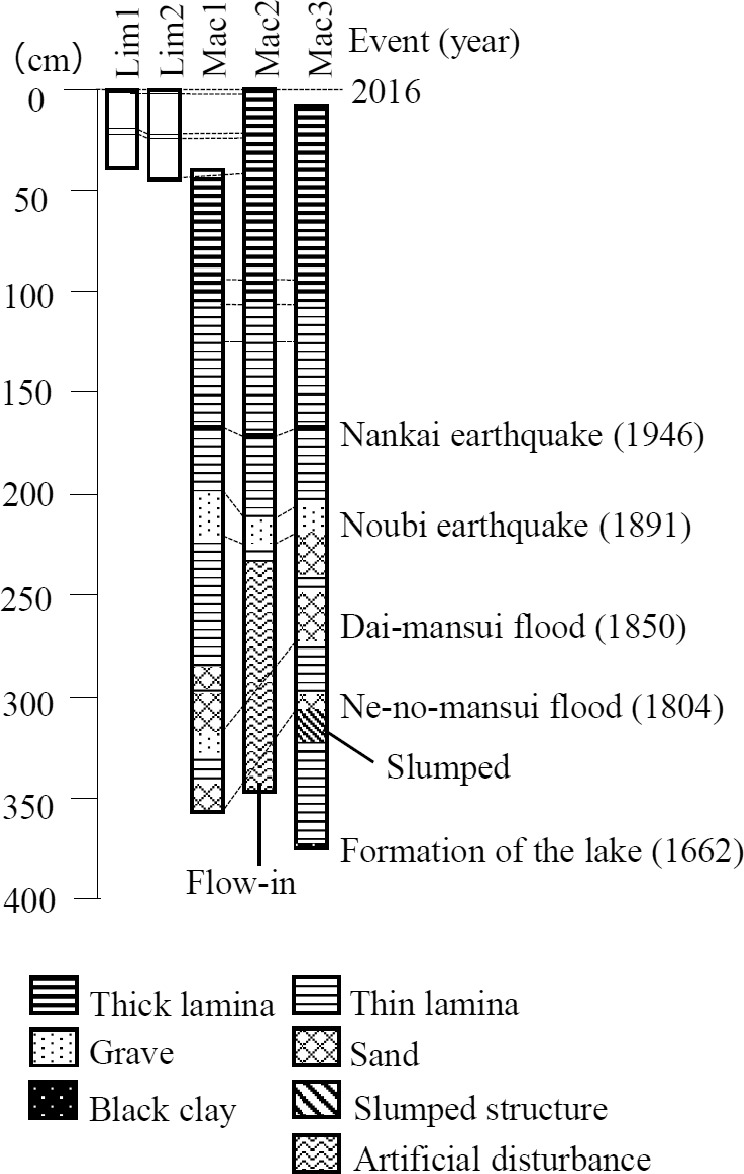
Stratigraphic correlation of sediment core samples taken from Lake Fukami‐ike

Lake Fukami‐ike (35°19′N, 137°49′) is located in Anan town, Nagano Prefecture, Japan (Figure 1). The lake is naturally formed and currently eutrophic with a maximum depth of 7.8 m and a surface area of 2.2 ha (Yagi et al., 2009). The lake was formed by a landslide triggered by an earthquake in 1662 (Table 1, Ueno, 1952), and environmental archeologists have succeeded in collecting sediment core samples that included the layer indicating the 1662 earthquake (Yamada et al., unpublished). A total of five lake sediment cores were collected from around the center of the lake on 6 and 7 October 2016 (Figure 1). Two short sediment cores (Figure 2), each ~35–42 cm long, were collected using a gravity corer (Limnos corer; Kansanen et al., 1991) with an internal diameter of 93 mm. Three long sediment cores (Figure 2), each ~306–360 cm long, were collected using a Mackereth corer (Mackereth, 1958) with an internal diameter of 65 mm. The two short and three long cores were sliced at 3‐cm and 10‐cm intervals, respectively. Then, these sliced samples were stored at 4°C in the dark. Each sample was mixed well, and then, 1 cm^3^ was measured for the wet weight (WW). Then, 1 cm^3^ was dried at 60°C for 48 hr to measure dry weight (DW). The ratio of WW to DW was calculated for each sample.

**Table 1:** Events preserved in the sediment core as key layers and historical records of fish introduction to and invasion of Lake Fukami‐ike

Year	Event	Fish introduction	Reference
1662	Lake formation		Ueno (1952), Kawakami et al. (2004)
1804	“Ne‐no‐mansui” flood		Matsushima (2000), Kawakami et al. (2004)
1850	“Dai‐mansui” flood		Matsushima (2000), Kawakami et al. (2004)
1891	Noubi earthquake		Matsushima (2000), Kawakami et al. (2004)
1945	Nankai earthquake		Matsushima (2000), Kawakami et al. (2004)
1960		*Hypomesus nipponensis*, *Carassius sp*. and *Cyprinus carpio* were introduced	Tanaka (1992)
~1974		*Micropterus salmoides* invaded	Sakurai and Watanabe (1974)
~2005		*Lepomis macrochirus* invaded	Kawanobe and Hosoe (2008)

Our sediment core samples were dated (Figure 2) based on their correlation with sediment core samples dated via ^14^C dating and annual lamination counting in previous studies (Ishihara et al., 2003; Kawakami et al., 2004) and the sedimentation rate calculated in previous studies (Kawakami et al., 2004; Yagi et al., 2009). The previously analyzed sediment cores and our working cores were correlated using lithological tie points, and the layers that represent the different events that were dated in previous studies (Table 1; Ishihara et al., 2003; Kawakami et al., 2004). We decided the correlation between long cores and short cores based on the shared layers (19 cm of Lim1, 21 cm of Lim2, and 25 cm of Mac2; 23 cm of Lim1, 25 cm of Lim2, and 28 cm of Mac2; 37 cm of Lim2 and 45 cm of Mac2; Figure 2). The sedimentation rate (cm/year) was calculated based on the dates estimated for the sediment layers and the thickness of the sediments between the dated layers. We integrated the information from subfossils, nutrients, and fossil pigments of each core sample by smoothing (see below).

### 2.2 Nutrient and fossil pigment determination

**Figure 3:**
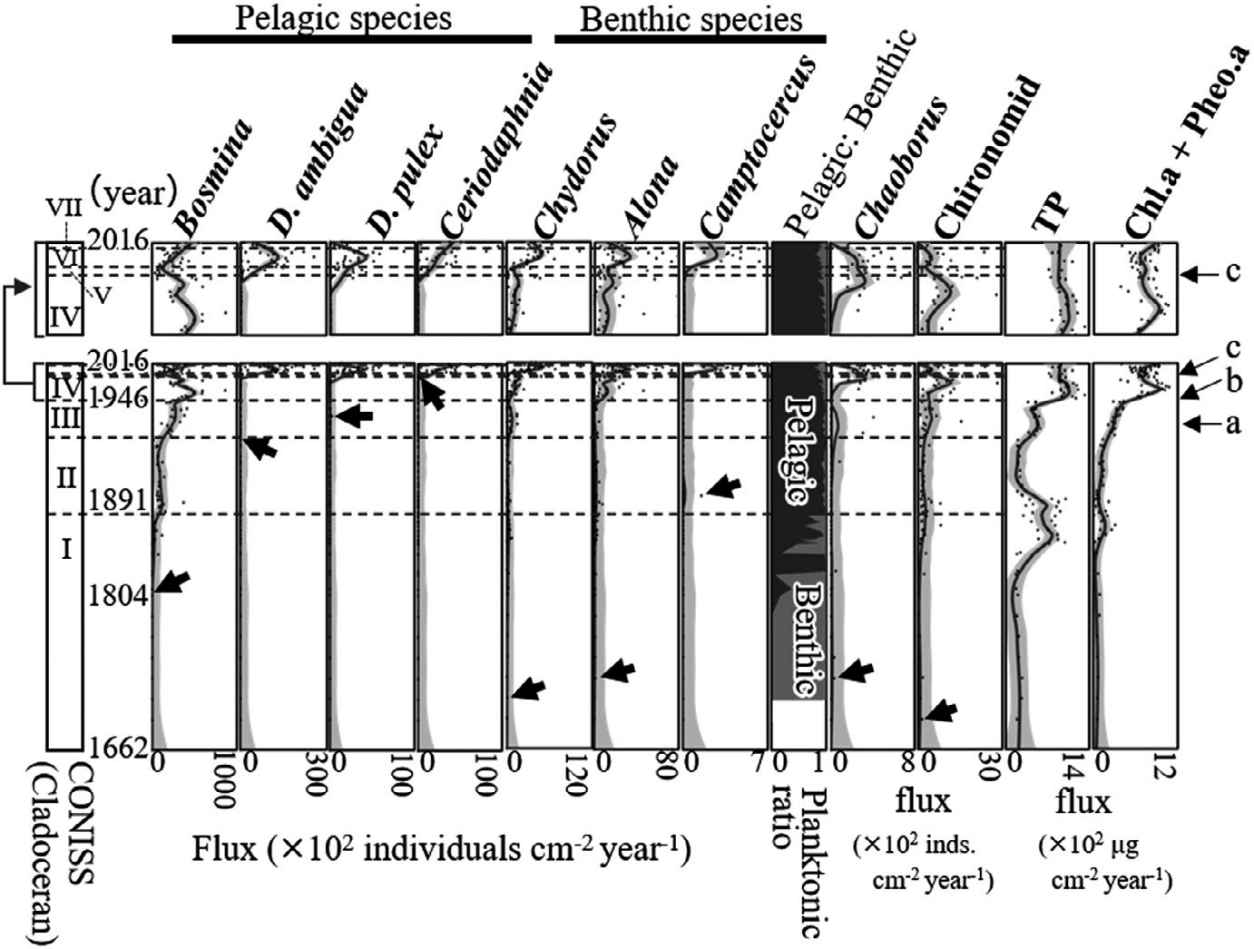
Dynamics of the cladoceran community and eutrophication process from the lake formation to the present. The lines represent the loess smoothed change in each species flux (span = 0.2) with 95% confidence intervals indicated by gray bands. Data points are the flux data of each species in each layer. Arrows indicate the year when each species first appeared in the sample. The far‐left column shows the periods of cladoceran community dynamics determined by CONISS. Arrows labeled with a–c indicate the timing of fish introduction: artificial introduction of *Hypomesus nipponensis* in 1960 (a); introduction of *Micropterus salmonids* in c. 1974 (b); and introduction of *Lepomis macrochirus* in c. 2005 (c). [Correction added on 13 January 2021 after first online publication: In Figure 3, *D. longispina* has been changed to *D. ambigua* in this version.]

We measured the concentration of total phosphorus (TP) and fossil pigment, chlorophyll a (Chl.a), and its derivative pheophytin‐a (Pheo.a), in every layer to estimate the nutrient increase process and test the bottom‐up effects on the cladoceran community. While testing the Chl.a, as it might possibly be degraded in the sediment, we also measured Pheo.a. Both Chl‐a and Pheo.a are commonly found in all algal taxa and were preserved in the sediments; therefore, we used them as proxies of phytoplankton abundance (Leavitt & Hodgson, 2002). The concentration of TP was measured using the molybdenum blue method (Murphy & Riley, 1962) after oxidization. Briefly, we weighed 0.5 g (WW) of sediment from each layer and dissolved it in 10 mL distilled water, then oxidized it with persulfate at 120°C for 60 min. After centrifugation (5 min)728.936 ×*g*, we separated the supernatant and measured absorbance at 880 nm to determine the concentration of TP with the calibration curve. The concentrations of Chl.a and Pheo.a were measured using the Lorenzen method (Lorenzen, 1967). We weighed 0.5 g (WW) of sediment from each layer and added 10 ml acetone; then, we mixed them in an ultrasonic bath. After leaving the samples for 24 hr at room temperature in the dark, we centrifuged them (728.936 ×*g*, 10 min). Then, we separated the supernatant and measured absorbance at 750 nm and 665 nm. We measured absorbance in the same way after the addition of two drops of 1 N hydrochloric acid. The concentration of Chl.a and Pheo.a was then calculated based on these absorbances. Concentration fluxes of TP, Chl.a, and Pheo.a (μg cm^−2^ year^−1^) were calculated from the concentration of TP, Chl.a, and Pheo.a (μg g WW^−1^) according to Kerfoot et al. (1999). We used the sum of Chl.a and Pheo.a fluxes as an indicator of phytoplankton abundance. As Chl.a flux was high when a peak of TP was observed (between period I and period II; Figure 3), the degradation of fossil pigments was assumed to be sufficiently negligible to reconstruct the long‐term changes.

Sediment P concentration may not reflect the P concentration in water due to sedimentary P mobility, causing sediment P to migrate to the sediment core surface influenced by redox chemistry and dissolved oxygen (Ginn et al., 2012). However, we observed the peak of TP not only near the surface but also in the middle of the sediment core (Figure 3). In addition, Chl.a + Pheo.a concentration, a proxy of phytoplankton abundance, showed similar peaks to TP (Figure 3). These peaks indicated that sedimentary TP concentration in this study probably exhibits a long‐term trend in bioavailable TP, even if they do not reflect the absolute concentration of TP in the water column.

### 2.3 Subfossils

We counted the cladoceran subfossils preserved in each sediment layer to examine the structure of the cladoceran community. We also counted chironomid subfossils as the major benthic invertebrates. In addition, we counted subfossils of *Chaoborus* larvae, selectively predated by fish, as a proxy for the abundance of planktivorous fish (Palm et al., 2011; Sweetman & Smol, 2006a, 2006b) to test top‐down effects on cladocerans. The *Chaoborus* specimens found in our samples were likely *C. flavicans* based on their morphology (Sweetman & Smol, 2006a, 2006b) and previous research on the studied lake (Nagano et al., 2014). Although *C. flavicans* can coexist with planktivorous fish as they exhibit diel‐vertical migration as a defensive strategy against fish predation, a previous study reported that *C. flavicans* tended to be more abundant in a fish‐less lake (Sweetman & Smol, 2006b), suggesting that the abundance of *C. flavicans* could reflect the degree of predation by planktivorous fish. Subfossils were counted according to the method of Korhola and Rautio (2001). Briefly, 1 g WW of sediment from each layer of all core samples was extracted and dissolved in 50 ml distilled water. Inorganic particles did not prevent observation, so we did not remove them. Then, 1 ml of the 50 ml sample was put into a 1‐ml Sedwick–Rafter chamber, and subfossils were counted twice (i.e., 2 ml in total) at a 200× agnification using an Olympus CX41 microscope. We counted and identified first 200 subfossils per layer and then confirmed whether other species were present in the rest of the 2 ml subsamples. If there were <200, then we counted all subfossils in the 2 ml subsamples. The cladoceran subfossils were identified with reference to the literature (Korosi & Smol, 2012a, 2012b; Sweetman & Smol, 2006a, 2006b; Tanaka & Makita, 2017), as were *Chaoborus* (Sweetman & Smol, 2006a, 2006b; Walker, 2001).

For the cladocerans identified as *Daphnia*, we further identified species based on the morphology of the postabdominal claw. We detected two types of postabdominal claws: one with distinct teeth and one without pectens. We also found two morphological types of ephippia that originated from *Daphnia pulex* and *Daphnia ambigua* in the same sediment core samples, and we confirmed that ephippia of *D. pulex* have mitochondrial DNA of *D. pulex* (Otake in prep). Furthermore, previous studies have reported *D. pulex* and *D. ambigua* in Lake Fukami‐ike (Nagano & Yoshida, 2020; So et al., 2015). Thus, we identified the postabdominal claw with distinct teeth as the *D. pulex* and those without pectens as the *D. ambigua*. If multiple body parts of one species were observed (i.e., head shield and carapace of *Bosmina longirostris*), we counted these separately, and then, the body part that was observed the most was used. The number of subfossils in each layer was converted to the number per g DW using the ratio of WW to DW of the sediment measured separately. Then, the flux of sedimented subfossils per year (number cm^−2^ year^−1^) was calculated as an index of the abundance of each species in each year using the sediment mass flux (g DW cm^−2^ year^−1^) following Kerfoot et al. (1999). For statistical analysis, we used only the species that accounted for >1% of the total number of cladocerans in at least one layer, as in Korhola (1999). In addition, we calculated the ratio of pelagic to benthic species subfossils in each layer to determine the succession from a benthic to a pelagic cladoceran community.

We attempted to assess the abundance of cyclopoid copepods that were not well preserved in the lake sediments owing to their soft carapace using a method based on the ratio of defensive head–carapace morphology of *Bosmina* according to Korosi et al. (2013). The specimens of *Bosmina* found in our samples were most likely to be identified as *B. longirostris* based on their morphology and the previous research on the studied lake (Suda et al., 2016; Tanaka, 1992; Ueno, 1952). *Bosmina longirostris* can exhibit morphological defenses against cyclopoid copepods. When cyclopoid copepods are present, *B. longirostris* exhibits long antennules (pellucida‐type), but when the copepods are absent, the antennules are curved (cornuta‐type) (Sakamoto et al., 2007). Thus, we can estimate the abundance of copepods from the subfossils of *B. longirostris*. We counted the subfossils of *Bosmina* by identifying the antennule, defensive pellucida, or nondefensive cornuta types. Then, we calculated the ratio of defensive pellucida‐type antennules to the total *B. longirostris* antennules for each layer of three sediment core samples (Lim1, Mac1, and Mac2).

Postabdominal claw length (PCL) of *Daphnia* species reflects body size (Hrbáček, 1969), and *Daphnia* body size decreases as the predation pressure from planktivorous fish increases (Jeppesen et al., 2002). Thus, PCL increases as predation pressure from planktivorous fish decreases (Amsinck et al., 2005; Perga et al., 2010). The PCL of *Daphnia* community was measured for each layer of two sediment core samples (Lim2 and Mac3) as an index of fish predation pressure following Korosi et al. (2011), using photographs taken by a digital camera (ARTCAM‐130MI) at 200× magnification (*n* = 1,097).

### 2.4 Statistical analysis

Although we used all data from all core samples for the following analyses of the cladoceran community and phytoplankton pigment, we omitted the samples Lim1 and Lim2, in which a sampling error occurred, for the measurement of TP concentration in the following analysis of TP. To determine the long‐term dynamics of the cladoceran community, TP, and phytoplankton pigment concentration, we smoothed the flux of cladoceran subfossils, TP concentration, and Chl.a + Pheo.a concentration by LOESS smoothing with the qplot and stat_smooth functions of the R package “ggplot2” (Wickham, 2016). We then examined the main changes or shifts in cladoceran community assemblages and trophic conditions using the constrained incremental sum of squares cluster analysis (CONISS, Grimm, 1987), with the broken stick model to assess the significance of CONISS‐delineated zones (Bennett, 1996). We conducted CONISS on all cladoceran subfossil data. These analyses were performed with the R packages vegan (Oksanen et al., 2017) and rioja (Juggins, 2017).

We used multivariate autoregressive models (MARs) (Ives et al., 2003) to evaluate whether changes in the cladoceran community could be caused by biotic interactions and eutrophication associated with changes in TP and phytoplankton pigment concentration. This analysis was performed using the R package MAR1 (Scheef, 2015). We used the fluxes of seven cladoceran subfossils as variables in the MAR model and added the TP and Chl.a + Pheo.a fluxes as exogenous covariables to assess the effect of eutrophication. In addition, to evaluate the effect of shifts in predators, we added the *Chaoborus* subfossil flux as a variable as we had done with the cladoceran subfossil fluxes.

First, we prepared the dataset using the “prepare data” function. We replaced the data of 0 flux as 1 and then log‐transformed all data. We standardized all data to have equal means and deviations for comparing between taxa. We fitted these data to the MAR model (Ives et al., 2003) by generating all possible models and then selecting the best‐fit model as the one with the lowest Akaike's information criteria (AIC). Then, we used bootstrapping (*n* = 500) on the best‐fit model to obtain 95% confidence interval (CI) for the coefficients in the model. Finally, we calculated the conditional *R* ^2^ for each taxon to evaluate the model's ability to predict the temporal changes in abundance. Model selection and estimation were performed using the “run.mar” function.

In addition, we examined the differences in *Daphnia* PCL between the periods using ANOVA and the post hoc comparison with the Tukey–Kramer test. All statistical analyses were performed using R version 3.5.2 (R Core Team, 2017), and significance was considered at *p* < .05.

## 3 RESULTS

Constrained clustering analysis CONISS (Grimm, 1987) was carried out on all cladoceran data. Seven periods were significantly identified for the cladoceran community dynamics (Figure 3, Figure S1): 1662 to early 1880 (period I), 1880 to early 1950 (period II), early 1950 to early 1980 (period III), early 1980 to early 2000 (period IV), early 2000 to mid‐2000 (period V), mid‐2000 to around 2014 (period VI), and around 2014 to 2016 (period VII).

Briefly, the long‐term dynamics of the cladoceran community suggested by the subfossils were as follows. First, the cladoceran community consisted only of benthic species in period I when both TP and Chl.a + Pheo.a were low. Then, small pelagic species of *Bosmina* appeared, and the ratio of pelagic taxa to benthic taxa increased in period II when the first peak of TP and Chl.a + Pheo.a. occurred. Then, the large cladocerans *Daphnia* appeared continuously from period V and increased in period VI when *Chaoborus,* which we used as a proxy of planktivorous fish, increased. Most recently, small *Bosmina* increased again in period VII when *Chaoborus* decreased.

In period I, the flux of cladoceran subfossils was entirely low. Benthic cladocerans, *Chydorus* and *Alona*, appeared and were continuously detected up to the surface of the sediments (i.e., 2016). In addition, before the cladoceran subfossils appeared, benthic chironomid larvae were observed. Until the later part of period I, TP flux and Chl.a + Pheo.a flux were low. From the end of period I to period II, small pelagic *Bosmina* appeared and increased. At this stage, the ratio of pelagic species to the total number of cladocerans began to increase (Figure 3, Figure S2). By the end of period II, *D. ambigua* was periodically but not continuously detected. Total phosphorus and Chl.a + Pheo.a fluxes were high at the beginning of period II but decreased by the end.

From period III to period IV, *Bosmina* further increased. Even though benthic *Chydorus* and *Alona* also increased, the proportion of pelagic species was still much higher than that of benthic species. Large cladocerans, such as the *D. pulex* and *D. ambigua*, occurred only in some layers in period III and not continuously. At the beginning of period III, the flux of TP and Chl.a + Pheo.a increased again. From period IV to period V, the cladoceran community became diverse, and *D. ambigua*, *D. pulex*, and *Ceriodaphnia* were continuously detected. Large cladocerans, *D. ambigua* and *D. pulex*, increased whereas small *Bosmina* decreased during this time. The flux of TP and Chl.a + Pheo.a increased and peaked during this time. After that, the flux of TP and Chl.a + Pheo.a remained at a high level up to the surface of the sediments (i.e., 2016).

In period VI, the two *Daphnia* peaked, although they tended to decrease at the end of the period. Conversely, small cladocerans, *Bosmina* and *Ceriodaphnia,* increased toward the end of period VI and period VII. In period VII, the large *Daphnia* continuously decreased (Figure 3, Figure S2).

*Chaoborus* larvae, an indicator of fish abundance, increased from the latter part of period IV to period V and decreased from period VI to period VII (Figure 3). This result indicated that planktivorous fish decreased from period IV to V and increased from period VI to period VII.

**Figure 4:**
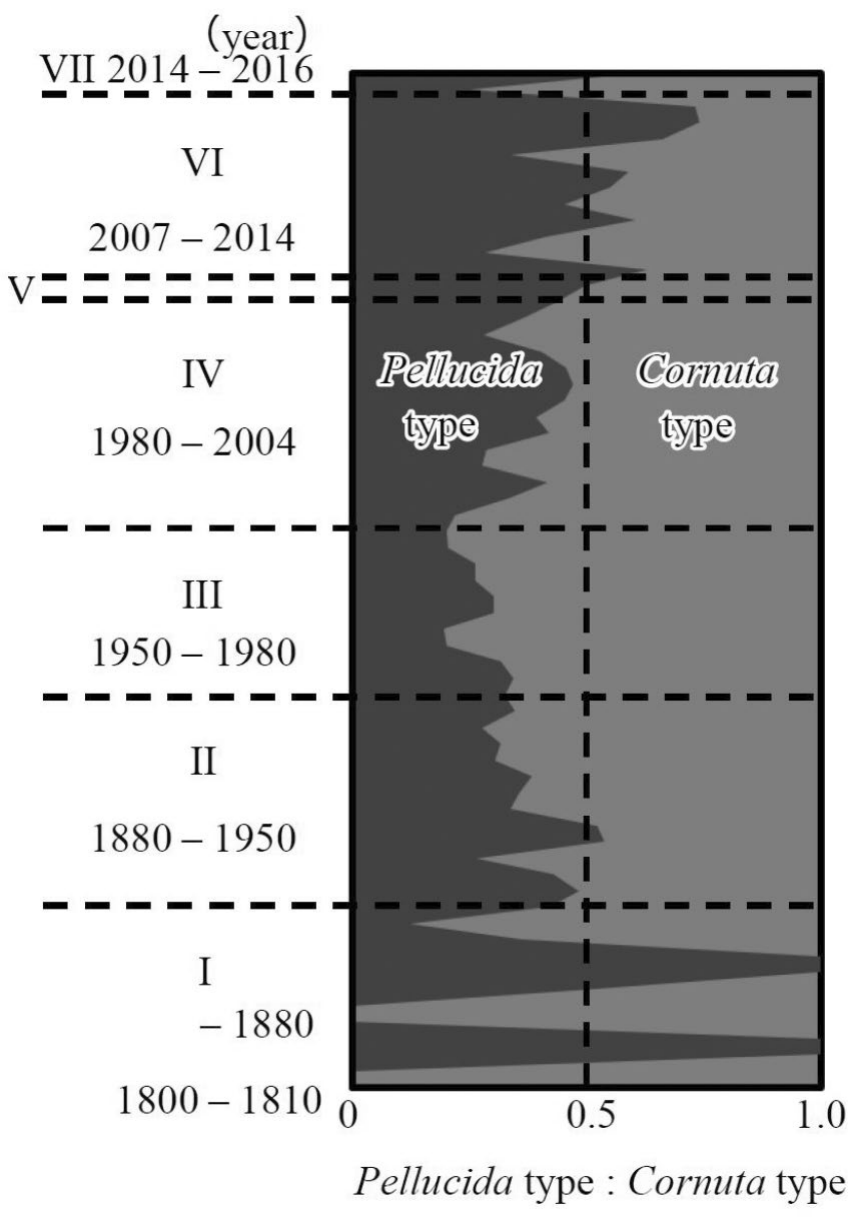
Changes in *Bosmina* antennule type shown as the ratio of pellucida‐type individuals to cornuta‐type individuals. The pellucida‐type antennule (dark gray) is a defense trait against cyclopoid copepods, whereas the other cornuta type (light gray) is a hooked antennule that cannot reduce predation risk from cyclopoid copepods

The ratio of the defended type (i.e., pellucida‐type) antennules of *B. longirostris*, a proxy of copepod community change, also showed long‐term changes (Figure 4). Cornuta type, which is the nondefended type, was more abundant from period II to period IV. Conversely, the layers in which the ratio of defended type exceeded 0.5 began to be detected after period V, although they were also observed in period I when the abundance of this species was very low (probably due to sampling error associated with low abundance).

**Figure 5:**
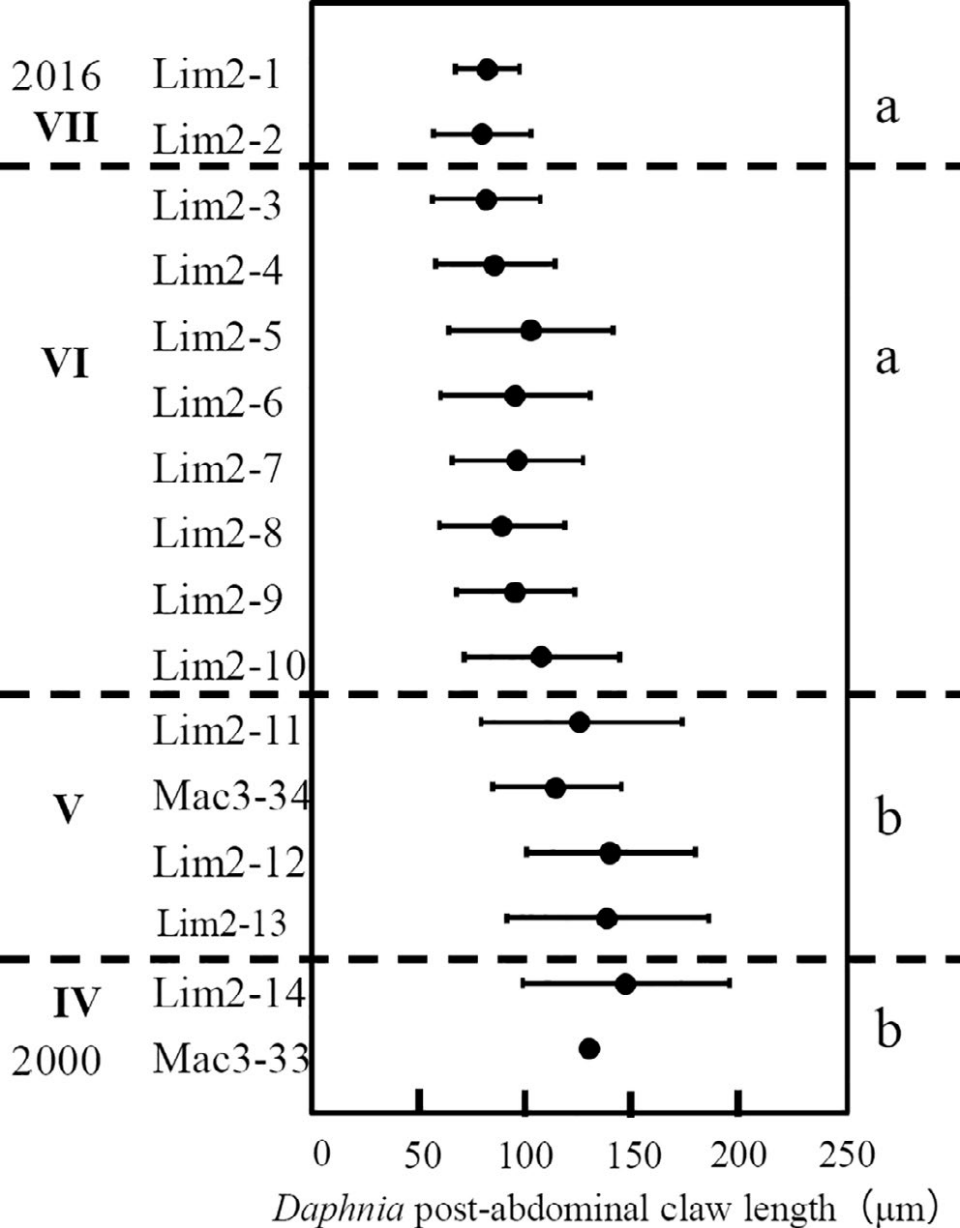
Changes in *Daphnia* postabdominal claw length (*n* = 1,097). Each plot shows the average length of the postabdominal claw from all *Daphnia* species in each layer. The bars show SE. IV to VII indicate the period of cladoceran community dynamics determined by CONISS. “a” and “b” are the results of the post hoc multiple comparison with the Tukey–Kramer test among CONISS periods (a‐b: *p* < .001)

*Daphnia* PCL was significantly different between CONISS periods based on ANOVA results (*p* < .001, Figure 5). The post hoc multiple comparison with the Tukey–Kramer test indicated that there were significant differences between periods VI and VII and periods IV and V (*p* < .001). In periods VI and VII, the PCL of *Daphnia* was shorter than that in periods IV and V.

**Figure 6:**
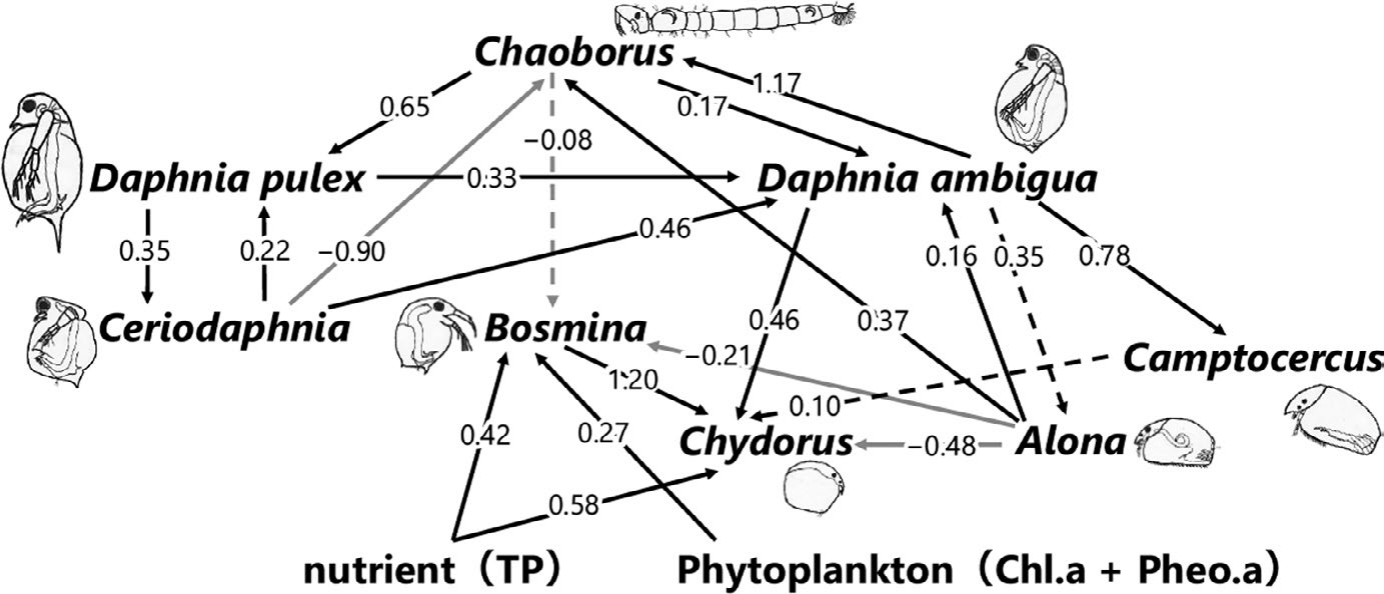
Bottom‐up effects, top‐down effects, and effects of interspecies interactions on the cladoceran community based on MAR model analysis. The coefficients were tested for significance by 500‐step bootstrap analysis (significant in bold). Black arrows indicate positive coefficients, and gray arrows represent negative coefficients. Solid arrows indicate significant coefficients, and broken arrows indicate nonsignificant coefficients based on a bootstrap analysis

The results of MAR indicated the following regarding the factors affecting the cladoceran community (Figure 6, Table 2). Chl.a + Pheo.a flux had a significant positive effect on *Bosmina*, and TP flux had a significant positive effect on both *Bosmina* and *Chydorus*. *Chaoborus* larvae positively affected the *D. ambigua* and *D. pulex*, whereas the negative effect of this predator on *Bosmina* was not significant, indicating that planktivorous fish negatively affected large *Daphnia* and but not small *Bosmina*.

**Table 2:** *R* ^2^ of the best‐fit multivariate autoregressive model for cladoceran community change in Lake Fukami‐ike

	Response							
	Bosmina	Daphnia ambigua	Daphnia pulex	Ceriodaphnia	Chydorus	Alona	Camptocercus	Chaoborus
*R* ^2^	0.64	0.91	0.91	0.85	0.90	0.49	0.58	0.58

Note

*R* ^2^ calculated with the bootstrapped model indicates how well the model predicts changes in density of each species from one step to the next.

## 4 DISCUSSION

In this study, we revealed the long‐term dynamics of the cladoceran community from lake formation onward and the change in both bottom‐up and top‐down effects on the cladoceran community. These results supported the hypothesis based on the previous studies about assembly and dynamics of the cladoceran community and temporal changes in controlling mechanisms as nutrient levels increase, including the replacement of the benthic with the pelagic community due to eutrophication (Bennion et al., 2015; Davidson et al., 2011; Jeppesen et al., 2011; Taylor et al., 2006) and the increased importance of top‐down effects with eutrophication (Davidson & Jeppesen, 2013; Jeppesen et al., 1997; McQueen et al., 1986). The cladoceran community consisted of benthic species under oligotrophic conditions in the lake formation period, which were replaced by pelagic species due to rapid eutrophication. These dynamics must be mainly controlled by bottom‐up effects. Under conditions in which nutrient levels became sufficiently high after rapid eutrophication, large *Daphnia* became established in the later part of period IV. In addition, high‐order consumers also became established at that time. As a result, the cladoceran community was mainly controlled by top‐down effects, initially affecting the establishment of large *Daphnia* and latter reducing cladocerans' body size.

### 4.1 Bottom‐up effects on cladocerans during the early periods

In the early stage of lake formation, TP and Chl.a + Pheo.a were low (Figure 3—period I), indicating that the lake was oligotrophic. In this period, only benthic cladocerans, *Chydorus* and *Alona*, were present, and the ratio of pelagic to benthic taxa was continuously low (Figure 3). In addition, benthic chironomids had already appeared (Figure 3). These results suggest that the benthic cladoceran community dominated under oligotrophic conditions in which the main primary producers were not phytoplankton (Bennion et al., 2015; Davidson et al., 2011; Jeppesen et al., 2011; Taylor et al., 2006). This finding supports the hypothesis that the benthic community first assembled during the early stages of the cladoceran community.

The present study also showed the cladoceran community changing from a benthic to a pelagic community in response to the phytoplankton becoming the dominant primary producer with increasing nutrient level (Bennion et al., 2015; Davidson et al., 2011; Jeppesen et al., 2011; Taylor et al., 2006). In Lake Fukami‐ike, rapid eutrophication has occurred twice (Figure 3). Fluxes of TP and Chl.a + Pheo.a increased around 1850 and 1950–1960, indicating eutrophication of the lake and an increase in phytoplankton. When eutrophication occurred around 1850, small pelagic *Bosmina* appeared and increased, eventually becoming dominant in the cladoceran community (Figure 3). This led to the replacement of the benthic cladoceran community with the pelagic community in the transition from period I to period II, which was revealed by the CONISS analysis (Figure 3). The first rapid eutrophication event could have caused these changes in the cladoceran community. The results of the MAR model were consistent with this; both the TP and Chl.a + Pheo.a fluxes positively affected *Bosmina* (Figure 6). This result agrees with that of previous studies reporting that *B. longirostris* increased as eutrophication proceeded (Gąsiorowski & Szeroczyńska, 2004; Ohtsuki et al., 2015). Furthermore, pelagic *Bosmina* did not have a negative effect on the benthic *Chydorus* and *Alona* according to the results of the MAR model analysis (Figure 6). Therefore, the replacement of the benthic community by the pelagic community seen in our study was possibly not due to competitive interactions with benthic species but rather to changes in primary producers, which both the pelagic and benthic taxa use.

Previous studies suggesting that the replacement by the pelagic community was due to eutrophication analyzed the change in the community before and after industrial eutrophication around 1950–1970 (Bennion et al., 2015; Davidson et al., 2011; Jeppesen et al., 2011; Taylor et al., 2006). In contrast, our study suggested that such replacement by the pelagic community occurred during the first rapid eutrophication event around 1850 before the second rapid eutrophication event, which was similar to those occurred in lakes worldwide (Saijo & Mitamura, 2016; Sakamoto, 1973; Schindler, 2006) (Figure 3). The first eutrophication was possibly caused by extensive agricultural activity combined with a natural disaster.

The land around Lake Fukami‐ike was most likely used as mulberry and rice fields in 1850 as shown in the earliest map of the area, published in 1908 (Topographic Map 50000 Geospatial Information Authority of Japan, 1908). In Anan town, chemical fertilizers have been widely used since the 1970s (Record of Anan town Compilation Committee, 1987). Before the 1970s, plant ash was used as a fertilizer, and thus, many grass fields were maintained to grow material for fertilizer, and wild fields were burned to generate ash (Record of Anan town Compilation Committee, 1987). These fertilizers may have been used to treat the mulberry and rice fields surrounding the lake. In 1850, a severe flood, called the “Dai‐mansui” flood, occurred around Lake Fukami‐ike (Matsushima, 2000). The layers representing this event were formed by the inflow of sediment from the surrounding area due to heavy rain (Figure 2, Kawasaki et al., 2004). Thus, eutrophication could have occurred through the fertilized soil flowing into the lake. Furthermore, nutrients accumulated in the lake sediments could be used by phytoplankton through biological decomposition (Keatley et al., 2011; Schindler, 2006); thus, a single notable influx of nutrients due to the flood could have supported phytoplankton growth for some years after the flood. Therefore, our study suggests that rapid eutrophication, even if not the recent industrial eutrophication event due to the inflow of chemical fertilizer and sewage water, can cause the same replacement of the benthic cladoceran community by the pelagic community.

When the second rapid eutrophication event occurred around 1950, pre‐established cladocerans: *Bosmina*, *Chydorus*, and *Alona* increased from period III to period IV (Figure 3). Like the replacement process, this increase in pre‐established cladocerans could have occurred due to eutrophication. The MAR model analysis showed that TP positively affected *Chydorus* and *Bosmina* (Figure 6). The results agree with those of previous studies that showed more abundant *Chydorus spaericus* and *B. longirostris* with increasing nutrient concentration and biological production (Luoto et al., 2008; Nevalainen & Luoto, 2013). Increasing nutrient concentration can promote zooplankton reproduction by increasing phytoplankton biomass (Vanni, 1987). The MAR model supported the effect of increased phytoplankton: Chl.a + Pheo.a positively affected *Bosmina* (Figure 6). In contrast, Chl.a + Pheo.a did not positively affect *Chydorus* (Figure 6). *Chydorus spaericus* can live in pelagic habitats and in littoral or benthic habitats (Fryer, 1968), as this species can associate with algal filaments (Fryer, 1968) and also feed on small phytoplankton (de Eyto & Irvine, 2001). Thus, *Chydorus* may have been influenced by increased TP with more production in benthic and pelagic habitats but not by Chl.a + Pheo.a, which represented only the pelagic food resources.

### 4.2 Top‐down effects on cladocerans during the later periods

After increases in nutrient levels from two eutrophication events, the relative importance of the top‐down effects on the cladoceran community increased in Lake Fukami‐ike. After eutrophication occurred, the cladoceran community diversified from around 2000 and became dominated by small species in more recent periods. *Daphnia* and *Ceriodaphnia* were continuously detected from later in period IV and became established since period V. Large *Daphnia* decreased, and small *Bosmina* and *Ceriodaphnia* increased during the most recent periods VI and VII (Figure 3). Since period V, TP, and Chl.a + Pheo.a fluxes did not change much (Figure 3), and the MAR model suggested that the cladoceran species, which became established in period V, were not significantly affected by either TP or Chl.a + Pheo.a (Figure 6). In contrast, *Chaoborus* larvae, a proxy of planktivorous fish abundance (Sweetman & Smol, 2006a; Palm et al., 2011), had a significant positive effect on the larger *D. pulex* and *D. ambigua*, which became established in period V (Figure 6). This result suggests that the planktivorous fish might have negatively affected *Daphnia* and that the change in the cladoceran community after period V might be relatively influenced by top‐down effects rather than bottom‐up effects.

The change in fish abundance, based on subfossils of *Chaoborus* larvae and the PCL of *Daphnia*, indicators of planktivorous fish abundance, supported this argument. The abundance of *Chaoborus* larvae and the PCL of *Daphnia* changed since the 2000s. When *Daphnia* increased from the end of period IV and period V, *Chaoborus* larvae also increased (Figure 3), and the PCL was longer than in periods VI and VII (Figure 5), suggesting that planktivorous fish were less abundant. In contrast, when small cladocerans were dominant from period VI to period VII, *Chaoborus* larvae decreased, and the PCL was shorter, suggesting that planktivorous fish were more abundant. These dynamics and the MAR model analysis indicated that the reduction in planktivorous fish had a positive effect on *Daphnia* (Figure 6), facilitating the sustainability of *Daphnia* populations and causing the diversification of the cladoceran community in periods IV and V. In contrast, the body size of the cladoceran community decreased because the increase in planktivorous fish reduced the *Daphnia* population and the small *Bosmina* increased from late in period VI to period VII. Increasing numbers of planktivorous fish might have reduced *Chaoborus* and released small cladocerans from predation risk, leading to the dominance of *Bosmina*. Positive effects of planktivorous fish on small *Bosmina* were also seen in the MAR model; *Chaoborus* larva negatively affected *Bosmina*, although the effect was not significant (Figure 6). While *Chaoborus* larvae are important predators of juvenile *Daphnia* (Havel & Dodson, 1984), our statistical analysis showed the effect of *Chaoborus* on *Daphnia* was positive (Figure 6). This suggests that the relative effect of predation from planktivorous fish on *Daphnia* could be greater than that from *Chaoborus*. These patterns in cladoceran and fish communities observed in Lake Fukami‐ike agree with the size‐efficiency hypothesis that planktivorous fish selectively prey on large cladocerans such as *Daphnia*, and invertebrate predators such as *Chaoborus*, and they less selectively prey on small cladocerans such as *Bosmina* (Brooks & Dodson, 1965; Miner et al., 2012). Invertebrate predators selectively prey on small cladocerans over larger ones (Brooks & Dodson, 1965; Leavitt et al., 1994). These results agree with those of previous studies that reported that both *Chaoborus* larvae and *Daphnia* tended to appear and increase simultaneously (Kerfoot, 1981; Palm et al., 2011), and *B. longirostris* tended to increase when *Chaoborus* larvae disappeared (Arcifa et al., 2015; Luoto et al., 2008). In addition, the results of an enclosure experiment that introduced *Chaoborus* larvae and planktivorous fish were consistent with these changes (Hanazato & Yasuno, 1989).

The fish community dynamics suggested by the *Chaoborus* larvae and the PCL of *Daphnia* agree with the results of previous studies indicating that increases in TP finally lead to a decrease in the relative abundance of planktivorous fish to piscivorous fish (Jeppesen et al., 1997). Historical records of fish invasions and introductions from external sources (Table 1) have significant implications for the observed changes in the cladoceran community. *Hypomesus nipponensis*, a planktivorous fish that selectively preys on large zooplankton (Chang et al., 2005; Makino et al., 2001), was artificially introduced in 1960 (Tanaka, 1992). The presence of the piscivorous fish *Micropterus salmoides* was first recorded in 1974, and then, *H. nipponensis* disappeared due to predation by *M. salmoides* (Shimoina Board of Education, 2009). In 2005, the omnivorous *Lepomis macrochirus*, which preys on zooplankton (Sakano & Yodo, 2004), was found in the lake (Kawanobe & Hosoe, 2008). After that, *L. macrochirus* increased greatly in abundance between 2005 and 2007 (Kawanobe & Hosoe, 2008) and has recently dominated the fish community of the lake (Takei, 2010).

In period III, when *H. nipponensis* was artificially introduced, *D. pulex* was first detected but immediately disappeared, indicating that it had failed to establish a sustainable population (Figure 3). Similarly, *D. ambigua* was detected in only one layer but not continuously during period III (Figure 3). In addition, *Chaoborus* larvae were found in only a few layers from that period (Figure 3). These results indicate high predation pressure from planktivorous fish on large zooplankton during period III. Then, after the invasion of piscivorous *M. salmoides*, *Chaoborus* larvae increased from the 1980s, most likely due to the release from *H. nipponensis* predation. *Daphnia* were also able to establish a sustainable population probably due to the decrease in *H. nipponensis* following *M. salmoides* predation. The changes in the cladoceran community recorded in our study agree with those observed in previous studies that reported *Daphnia* becoming dominant after the introduction of piscivorous fish (Leavitt et al., 1994) and that the dominant taxon switched from *Daphnia* to *Bosmina* after the increase in planktivorous fish (Perga et al., 2010). Overall, our research demonstrated that changes in predator composition caused by human activity could significantly affect cladoceran community dynamics.

In the present study, *Daphnia* became established under conditions in which sufficient nutrients already existed in the lake after the second eutrophication (Figure 3). This result suggests that eutrophication relieves *Daphnia* from food limitations and that it could enhance the relative importance of top‐down effects on *Daphnia* and the cladoceran community. Increased numbers of *Daphnia* under high‐nutrient conditions were observed in some prior studies, including observation and mesocosm experiments (e.g., Declerck et al., 2007; George, 2012). For example, Straile and Geller (1998) showed that *Daphnia* biomass increased under a change from oligotrophic to mesotrophic status and mesotrophic to eutrophic status by comparing *Daphnia* biomass during three periods in Lake Constance: oligotrophic (1920s), mesotrophic (1950s–1960s), and eutrophic (1980s–1990s).

In addition, we tried to examine the dynamics of cyclopoid copepods, predators of small zooplankton, based on the changes in *Bosmina* antennule type (Figure 4). During periods VI and VII, when small species dominated the cladoceran community, the ratio of defended (i.e., pellucida‐type) *Bosmina* increased (Figure 4). This result suggests that cyclopoid copepod might have increased since period VI, supporting the argument that high nutrient levels can maintain the presence of high‐level consumers.

## 5 CONCLUSION AND FUTURE STUDIES

We observed the long‐term community dynamics of cladocerans from the time the lake was formed. Our results suggest that, under the early oligotrophic conditions, the cladoceran community consisted of a benthic community, which was replaced by a pelagic community due to eutrophication. Later, further eutrophication allowed high‐order consumers to establish, and the cladoceran community might have become controlled by top‐down effects. In the present study, we focused on cladocerans and evaluated phytoplankton change based on fossil pigments only. Thus, if we could analyze the subfossils of other taxa, including phytoplankton, we would be able to reveal the mechanisms underlying the bottom‐up and top‐down effects in more detail. Ishihara et al. (2003) analyzed planktonic diatoms in both light‐colored laminae and dark‐colored laminae to determine whether each laminae couplet represented an annual varve in Lake Fukami‐ike. However, since this previous study focused only on planktonic diatoms, we could not compare the eutrophication process indicated by diatoms and our TP and fossil pigment analyses or analyze the relationship between changes in the cladoceran and diatom communities in detail. However, Ishihara et al. (2003) found that *Aulacoseira* spp., reported by several studies as replacing and dominating benthic species under eutrophic conditions (e.g., Doig, Schiffer, & Liber, 2015), were abundant around 1,890 (Ishihara, unpublished), which agrees with our finding that the first rapid eutrophication was occurring at that time. Analyzing the relationship between cladoceran and other taxa such as phytoplankton and microbes is a topic for future study. Also, accumulating more paleolimnological datasets like those obtained by the present study should provide a more general understanding of the community succession process and its underlying mechanisms, which would be of much interest to researchers in limnological ecology.

## CONFLICT OF INTEREST

None declared.

## AUTHOR CONTRIBUTIONS

**Yurie Otake:** Data curation (lead); funding acquisition (equal); investigation (lead); writing–original draft (lead); writing–review and editing (lead). **Hajime Ohtsuki:** Data curation (supporting); investigation (equal); methodology (supporting); validation (supporting). **Jotaro Urabe:** Investigation (supporting); supervision (supporting); validation (supporting). **Shigeko Kimura:** Investigation (supporting); validation (supporting). **Kazuyoshi Yamada:** Data curation (supporting); investigation (supporting); validation (supporting). **Takehito Yoshida:** Funding acquisition (equal); investigation (equal); supervision (lead); validation (equal).

## Supporting information

Supplementary Material

## Data Availability

The data used to support the findings of this study are archived in figshare repository: https://doi.org/10.6084/m9.figshare.13182404.v3.
