# Supplementary material for: Long‐term dynamics of a cladoceran community from an early stage of lake formation in Lake Fukami‐ike, Japan
Source: Ecol Evol. 2020 Dec 24;11(3):1240–53. doi: 10.1002/ece3.7112 (PMC7863664; doi:10.1002/ece3.7112)

Supporting Information

Figure S1. A. Cluster plot from the CONISS analysis for the cladoceran community. B. Results of the broken stick model to determine the number of significant groups in CONISS analysis (Red line represents the sum of squares with broken stick model. Black line represents the sum of squares with CONISS analysis).


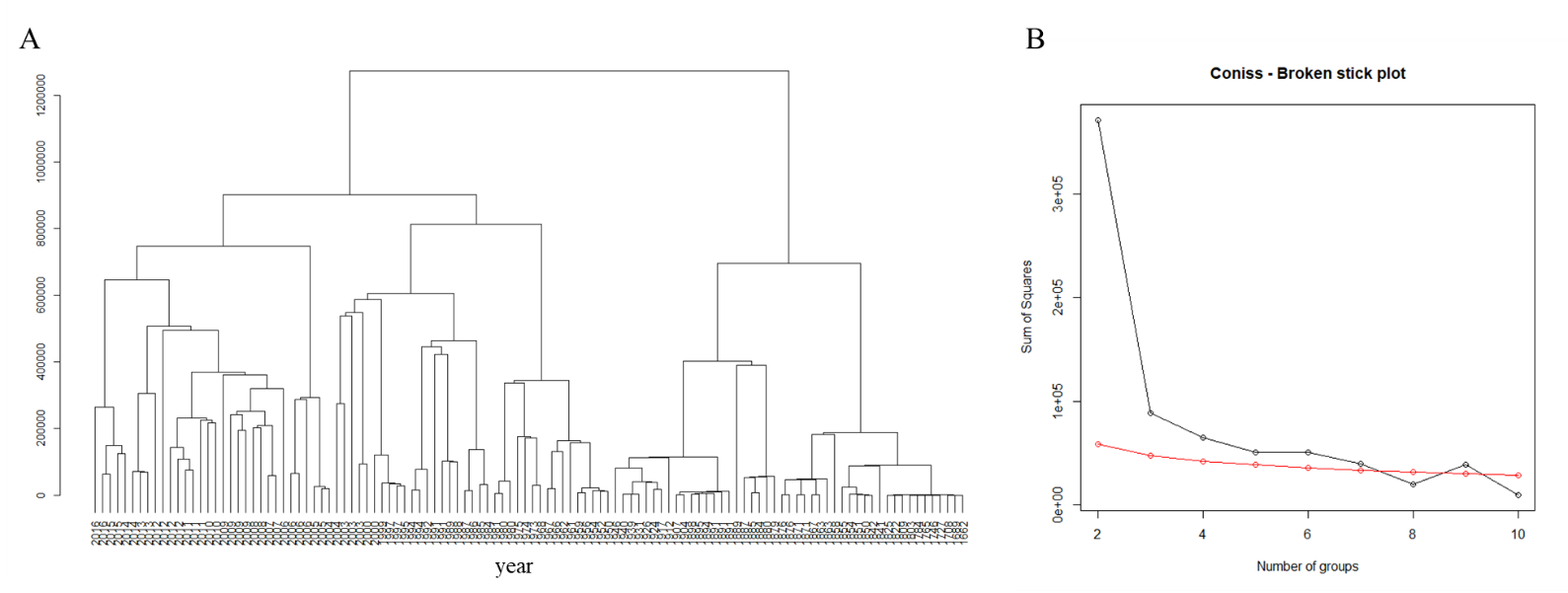


Figure S2. Percentage abundance of subfossil cladocerans among the total fossil content.

CONISS results are the same as in Figure 3.


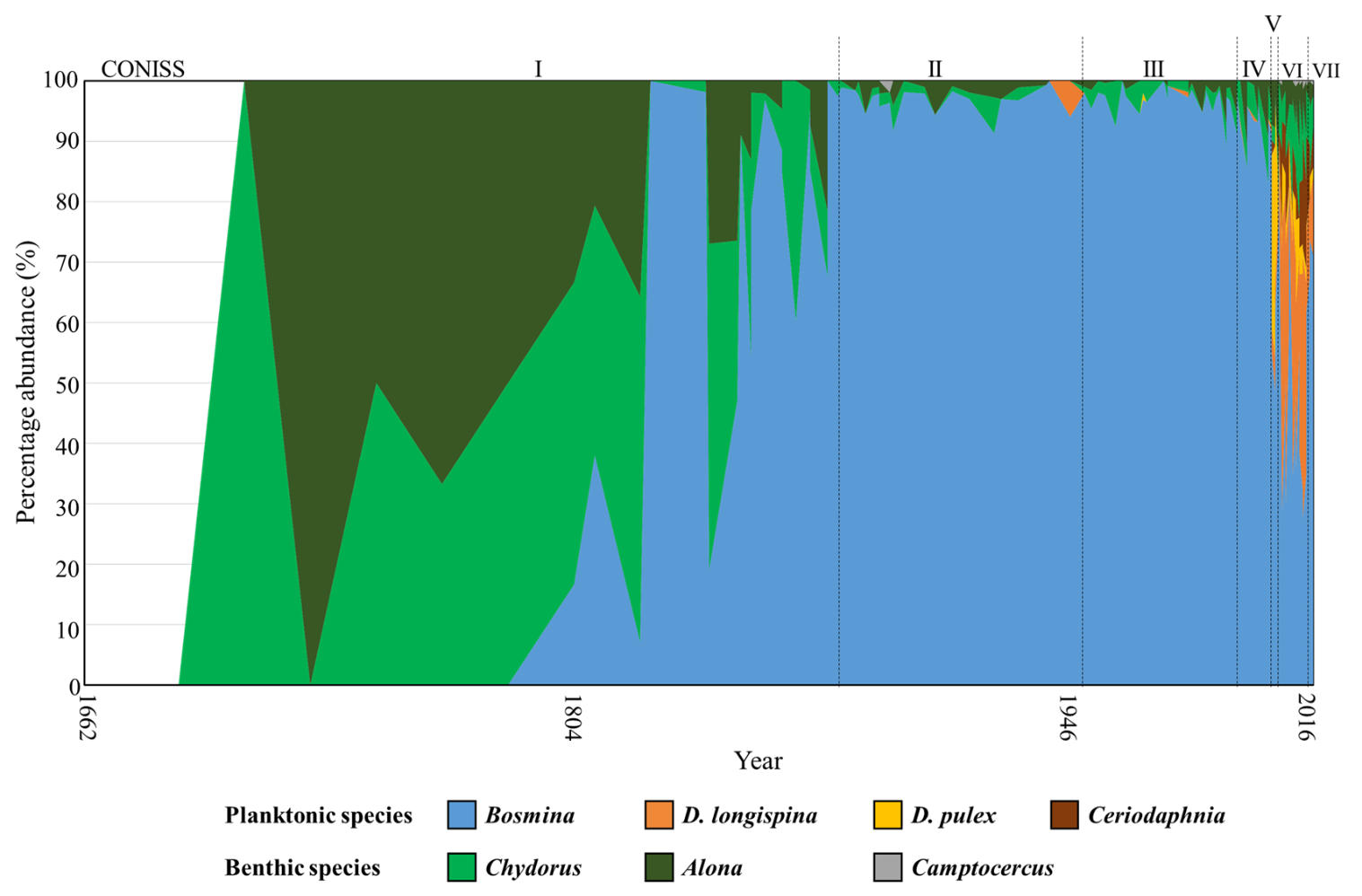

Supplement: Supplementary file 1 — Supplementary Material [file ECE3-11-1240-s001.docx]
